# The interfacial pH of acidic degradable polymeric biomaterials and its effects on osteoblast behavior

**DOI:** 10.1038/s41598-017-06354-1

**Published:** 2017-07-28

**Authors:** Changshun Ruan, Nan Hu, Yufei Ma, Yuxiao Li, Juan Liu, Xinzhou Zhang, Haobo Pan

**Affiliations:** 1 Center for Human Tissue and Organs Degeneration, Institute of Biomedicine and Biotechnology, Shenzhen Institutes of Advanced Technology, Chinese Academy of Sciences, Shenzhen, Guangdong 518055 China; 2 Key Renal Laboratory of Shenzhen, Department of Nephrology, Shenzhen People’s Hospital, The Second Clinical Medical College of Jinan University, Shenzhen, Guangdong 518020 China; 3 Department of Biochemistry and Molecular Biology, Southwest Medical University, Luzhou, Sichuan 646000 China

## Abstract

A weak alkaline environment is established to facilitate the growth of osteoblasts. Unfortunately, this is inconsistent with the application of biodegradable polymer in bone regeneration, as the degradation products are usually acidic. In this study, the variation of the interfacial pH of poly (D, L-lactide) and piperazine-based polyurethane ureas (P-PUUs), as the representations of acidic degradable materials, and the behavior of osteoblasts on these substrates with tunable interfacial pH were investigated *in vitro*. These results revealed that the release of degraded products caused a rapid decrease in the interfacial pH, and this could be relieved by the introduction of alkaline segments. On the contrary, when culturing with osteoblasts, the variation of the interfacial pH revealed an upward tendency, indicating that cell could construct the microenvironment by secreting cellular metabolites to satisfy its own survival. In addition, the behavior of osteoblasts on substrates exhibited that P-PUUs with the most PP units were better for cell growth and osteogenic differentiation of cells. This is due to the hydrophilic surface and the moderate N% in P-PUUs, key factors in the promotion of the early stages of cellular responses, and the interfacial pH contributing to the enhanced effect on osteogenic differentiation.

## Introduction

Osteoblasts play important roles in initial bone construction and later bone remodeling processes^1–3^. Accordingly, it has attracted much interest to accurately deliver, transport, fasten and functionalize osteoblasts by seeding on specific surfaces to stimulate bone formation, including ceramics^4^, polymers^5, 6^ and metals^7^. It is reported that the acid-base equilibrium plays an important role in influencing the behavior of osteoblasts, and thus affects the bone remodeling process^8–11^. Hench *et al*. verified that a weak alkaline environment stimulated the spontaneous formation of an apatitic layer on the surface of 45S5 Bioglass^12^, which was related to evaluating the osteoinduction ability of the implanted materials for bone regeneration *in vitro*. Meanwhile, our previous study indicated that a localized pH change significantly affected the proliferation and alkaline phosphatase (ALP) activity of osteoblasts^13^. We further demonstrated that the weakly alkaline pH of borosilicate showed the potential to stimulate osteoblast viability and activity, thus further facilitating apatite nucleation^14^. In addition, Liu *et al*. monitored the microenvironment pH of a series of alkaline biodegradable implant materials by a pH microelectrode *in vivo*, and confirmed that alkaline biodegradable materials generated an *in vivo* microenvironment pH which was higher than the normal physiological value, revealing promising healing effects in the context of osteoporotic bone defects^15^. In particular, the release of alkaline ions to generate a pH of ~8.5 on the material surface not only stimulates the nucleation of calcium phosphates, but also facilitates the proliferation of osteoblasts^16^. Efforts to increase the surface pH to design and make a specific material to stimulate new bone formation are therefore attractive^13, 15, 17–19^.

Unfortunately, such a hypothesis is inconsistent with the application of biodegradable polymers, as the degradation products are usually acidic^20, 21^. Even though biodegradable polymers have been widely used as bone substitutes, it is still not known exactly how this happens^22, 23^. The acidic environment caused by acidic degradation products may slow down the proliferation of osteoblasts. A comprehensive study in polymer systems is therefore necessary.

Most notably, polyester-based polymers, such as polylactide (PLA)^22^, polyglycolide (PGA)^24^, and polycaprolactone (PCL)^5^, are widely used as bone substitutes due to their good biocompatibility, ease of processing, appropriate mechanical properties, and biodegradability. However, the release of acidic products may acidify the microenvironment and thus lower the local pH, although this is thought to be buffered by the blood. Interestingly, a previous study has shown a significantly higher pH on a bioglass surface than in the bulk blood, due to the release of alkaline ions in an OVX rat model^15^. Here, the surface provides the initial requirement for osteoblast attachment and proliferation. Obviously, a local pH shift will determine the activity of cells, but the dynamic investigation of the interfacial pH is absent from most previous research.

**Figure 1:**
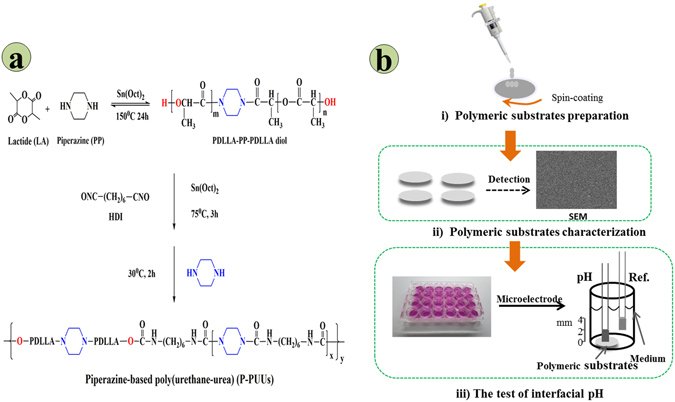
The synthetic route and structure of P-PUUs (**a**), and the flow chart of formation and the test of interface pH of the polymeric substrates (**b**).

This study was therefore carried out to investigate the impact of interfacial pH of acidic degradable polyester-based biomaterials on the growth of osteoblasts, while poly (D, L-lactide) (PDLLA) and piperazine-based polyurethane ureas (P-PUUs) were designed with various interfacial pH as examples of acidic degradable polymers for bone regeneration (Table 1). Previously^6, 21, 25^, P-PUUs have been prepared by us with poly (D, L-lactic acid) diol, hexamethylene diisocyanate (HDI) and piperazine (PP) as the chain extender (Fig. 1a). The amount of PP in P-PUUs can be quantitatively altered by regulating the molar ratio of PDLLA diol: HDI: PP in the reaction feed. This demonstrated that P-PUU was an ideal scaffold for bone regeneration with appropriate mechanical properties^25^, shape memory behavior^25^, and controllable degradation^6, 21^. Herein, the substrates of PDLLA and P-PUUs with gradient PP uniformly covered on glass slides were obtained using a spin-coating technique (Fig. 1b). The surface morphology, static water contact angles, and nitrogen content of the polymeric substrates were detected. Meanwhile, investigations of the interfacial pH of polymeric substrates with or without osteoblasts were performed by a known flat membrane microelectrode method. In addition, the behavior of osteoblasts on these substrates was evaluated to clarify the role of the interfacial pH of acidic degradable biomaterials for bone regeneration.

**Table 1:** Polymers used in this study.

Polymer^a^	Diol:HDI:chain extender^b^	*Mn*(10^4^)^c^	PDI^c^	N%^d^	Contact angle (°)
PDLLA	—	5.843	1.14	—	92.7 ± 1.2
P-PUU-1	1:1.1:0.1	5.785	1.15	0.373%	88.1 ± 1.1
P-PUU-2	1:1.2:0.2	6.432	1.23	0.559%	86.5 ± 0.9
P-PUU-3	1:1.4:0.4	7.432	1.36	0.828%	84.8 ± 1.3

^a^Sample code: see text. ^b^The molecular weight of PDLLA diol was 3592, measured by ^1^H NMR. ^c^Measured by gel permeation chromatography. ^d^Tested by XPS (1).

## Results

### Characterization of polymeric substrates

**Figure 2:**
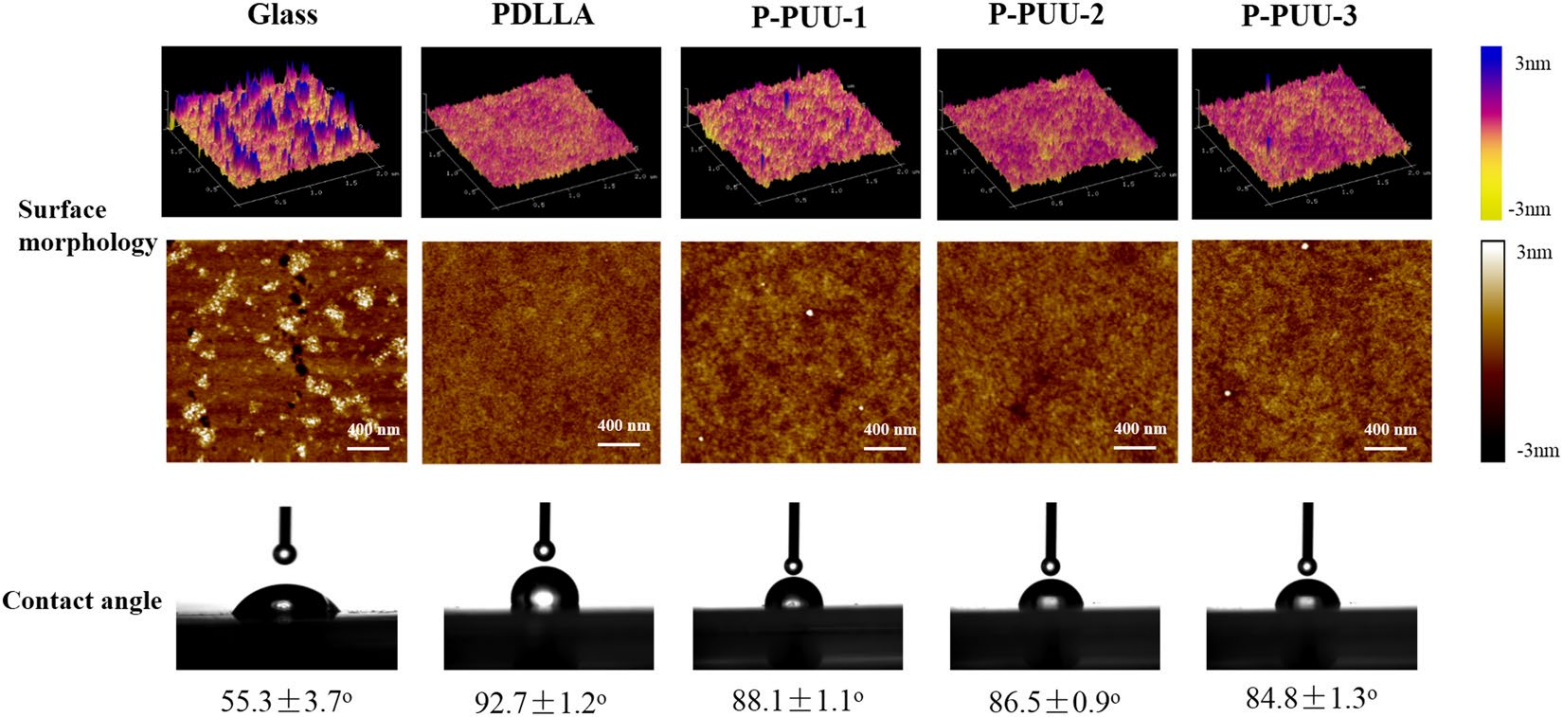
The surface morphology and static water contact angle of the polymeric substrates.

**Figure 3:**
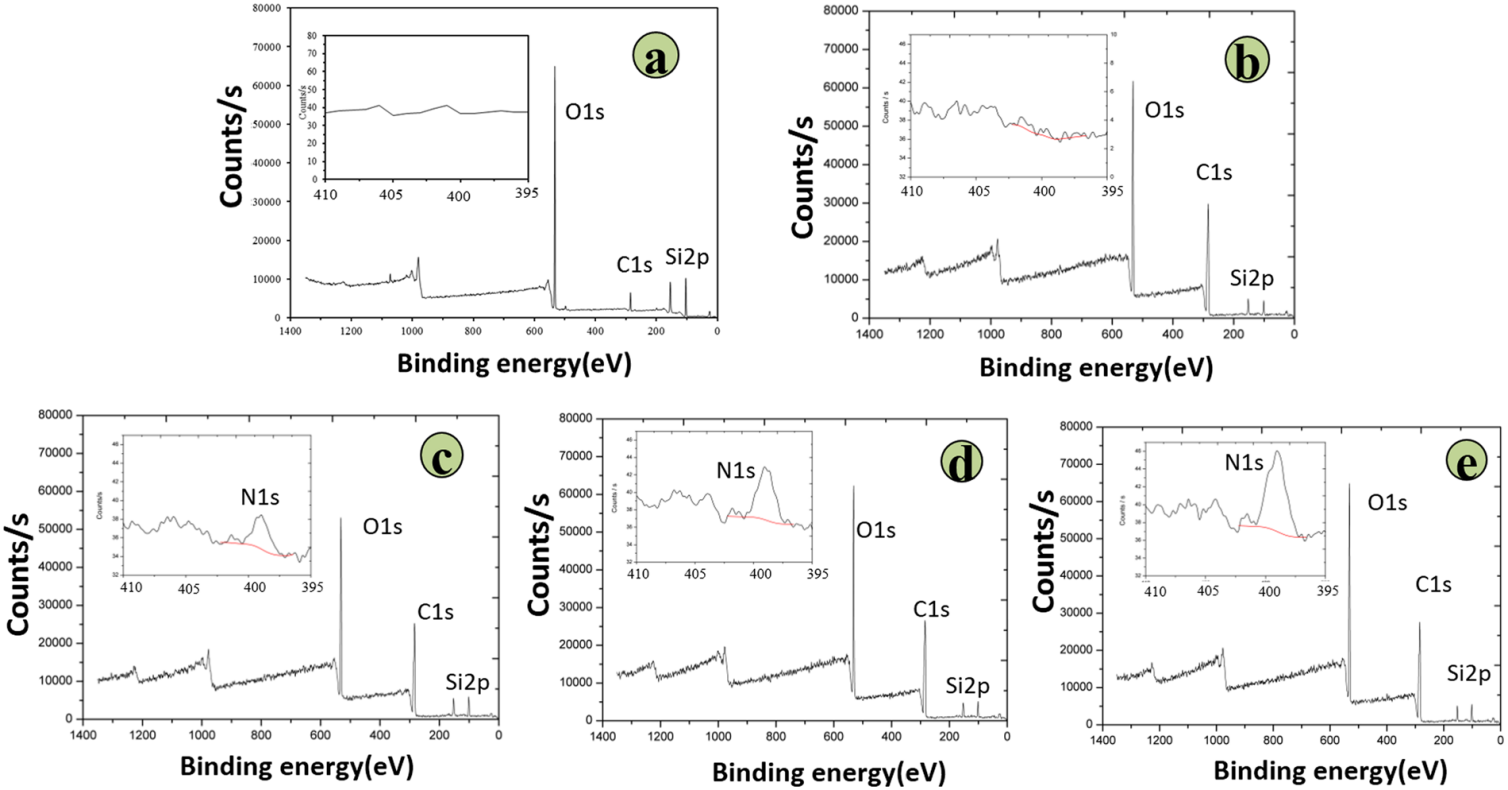
The amount of nitrogen in the surface of polymers tested by XPS: (**a**) Glass 0; (**b**) PDLLA 0; (**c**) P-PUU-1 0.373%; (**d**) P-PUU-2 0.559%; (**e**) P-PUU-3 0.828%.

The surface morphologies and static water contact angles of the substrates are shown in Fig. 2. It was observed by AFM analysis that the surface of glass group was rougher than those of polymeric substrates in the micron-nanometer range while all polymeric substrates revealed similar surface morphology. Due to its rough surface, the glass surface demonstrated with more hydrophilicity (55.3 ± 3.7°) compare to the surface of polymeric substrates and with increasing PP content, the static water contact angles of P-PUUs and PDLLA decreased from 92.7 ± 1.2° for PDLLA to 84.8 ± 1.3° for P-PUU-3, suggesting that their surface hydrophilicity had been improved by introducing PP-related bonding. Moreover, the surface chemistry elements of the glass control and polymeric substrates were characterized by XPS as shown in Fig. 3. The XPS pattern of glass (Fig. 3a) revealed the main composition of commercial glass slides we used as positive control was silica glass. The peaks at 532.8 eV and 284.8 eV were attributed to C1s and O1s of PDLLA (Fig. 3b) and P-PUUs (Fig. 3c–e), whereas the peaks at 152.8 eV and 101.8 eV were attributed to Si2p of the silicon substrate, which was used as a support for XPS detection in this study. The N1s peaks at 399.8 eV were observed in the inset figures of the P-PUUs, but not in the inset figure of glass and PDLLA, due to the addition of the PP and HDI units to the P-PUUs. Meanwhile, the N% in the P-PUUs was improved by increasing the amount of PP in the P-PUUs. The N% in P-PUU-1, P-PUU-2 and P-PUU-3 was 0.373%, 0.559%, and 0.828%, respectively.

### Interfacial pH

**Figure 4:**
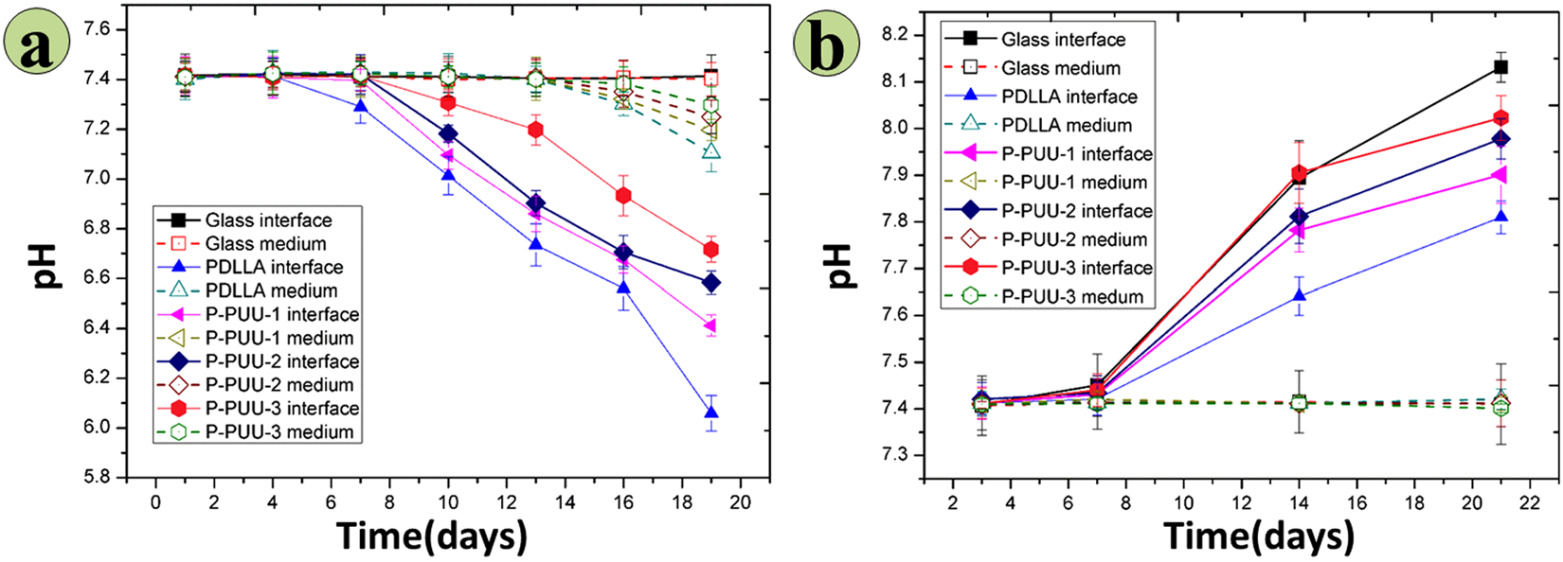
The interfacial micro-pH of substrates cultured in medium, and the pH value of the medium: (**a**) without osteoblasts (at 1, 4, 7, 10, 13, 16 and 19 days, and after 1 day of the renewed medium point); (**b**) with osteoblasts (at 3, 7, 14, and 21 days, and after 1 day of the renewed medium point).

As expected from the XPS results and previous reports^6^, regulating the amount of PP in the P-PUUs can change the N% on the surface of the P-PUUs, and the degradation behavior, which would directly impact the interfacial pH of P-PUUs. Figure 4 illustrates the interfacial pH of the polymeric substrates and the glass control cultured in medium without or with osteoblasts. In Fig. 4a, the pH variations for the cultured medium (3 mm above the surface) were minor, and the values were mostly around pH 7.4. The trend seen with the interfacial pH of glass was similar to that of the cultured medium pH with glass, due to the lack of degradation. Nevertheless, the interfacial pH of the polymeric substrates as a function of the cultured time decreased, and as the N% increased, the variations of interfacial pH became milder. Particularly, after culturing for 7 days, in every sample of polymers, the interfacial pH was lower than its medium pH.

The variations in the interfacial pH and medium pH of substrates with osteoblasts are presented in Fig. 4b. Interestingly, a completely different tendency for the interfacial pH emerged between polymeric substrates without cells (Fig. 4a) and with cells (Fig. 4b). It was found that the interfacial pH values of all the substrates with osteoblasts as a function of the cultured time increased and surpassed their medium pH. These variations of interfacial pH became more apparent with an increase in the N%. At 14 and 21 days of culture, the interfacial pH of every sample was higher than its medium pH. In particular, for P-PUU-3 at 21 days, the interfacial pH values reached 8.13, approaching the optimum value for osteoblast growth according to a previous study^13^. Moreover, the highest interfacial pH after culturing for 21 days with cells was that of the glass substrate, due to the lack of degradation.

### Cell morphology and proliferation

**Figure 5:**
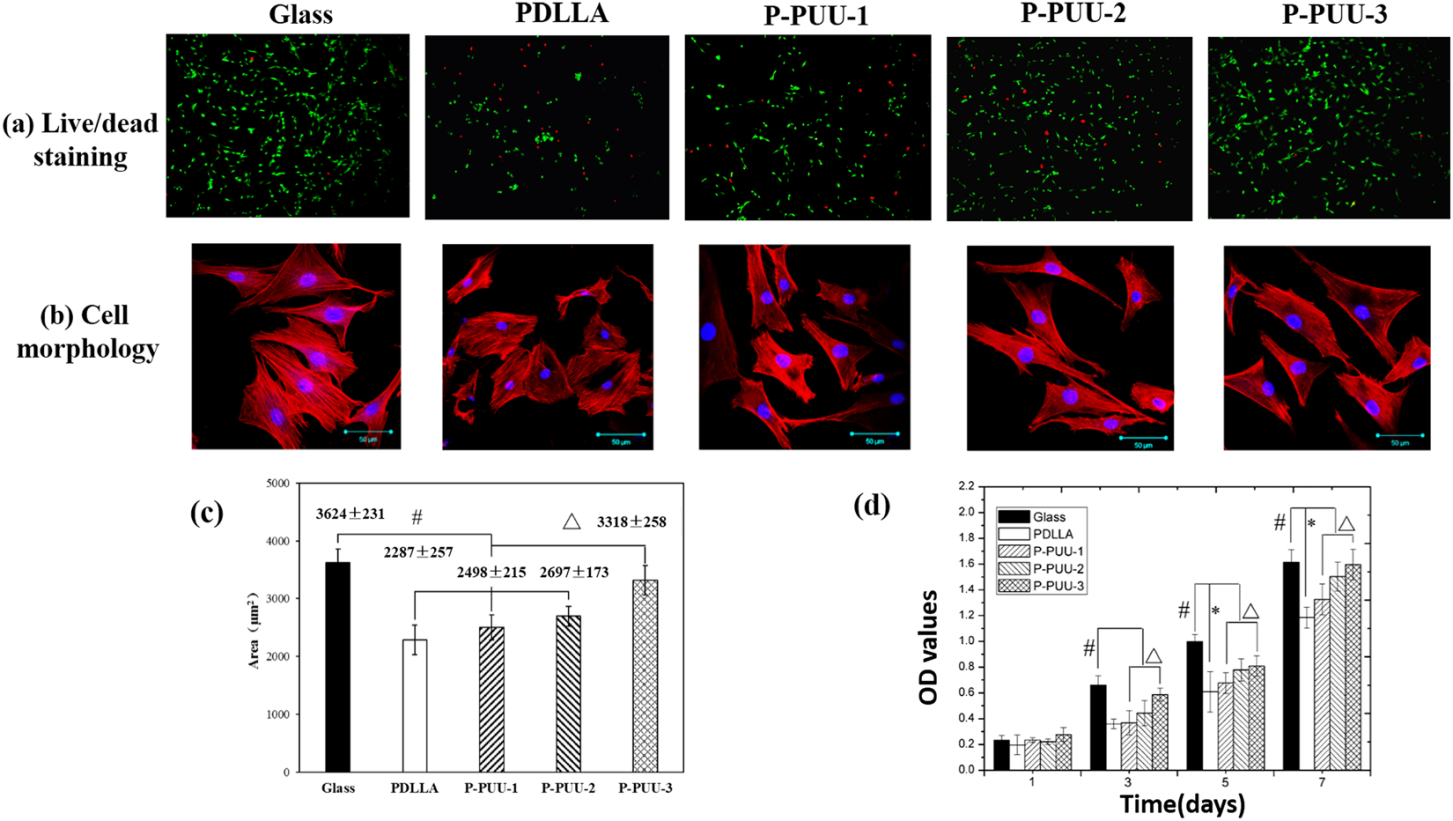
Morphology and proliferation of osteoblasts on different substrates: (**a**) Live/dead staining for cell after seeding of 24 h, green fluorescence indicating cells alive while the red visualizing dead cells; (**b**) Morphology and of osteoblasts on glass, PDLLA, P-PUU-1, P-PUU-2, and P-PUU-3 by CLSM at 24 h; (**c**) cell spreading areas (n = 200) after seeding of 24 h; (**d**) Cell proliferation by MTT at 4, 7, 14 and 21 days (# means P < 0.05, compared with glass groups; * means P < 0.05, compared with PDLLA groups; Δ means P < 0.05, compared with P-PUUs groups).

The cell viability and morphology of the substrates is an important factor, and can reflect the initial interaction between cells and biomaterials^26^. The viability and morphology of osteoblasts on various substrates for 24 h of culture were detected as shown in Fig. 5. Live/dead staining (Fig. 5a) revealed that almost no red dead cell was observed on glass substrates, and there were fewer red dead cells on P-PUUs than those on PDLLA, suggesting the better primary compatibility of ISO-PUs than PDLLA. Moreover, the viability could be improved with increasing the PP amount in the P-PUUs. This was further confirmed by the results of morphology of osteoblasts (Fig. 5b,c). The cell nucleus and actin cytoskeleton were visualized by staining blue and red, respectively. The cells on P-PUUs films spread better than those on PDLLA films (2287 ± 257 µm^2^) and the cell areas increased from 2498 ± 215 µm^2^ to 3318 ± 258 µm^2^ with the increase of hard segments content from 1.0:1.1:0.1 to 1.0:1.4:0.4.

Proliferation of the osteoblasts on different substrates is shown in Fig. 5d. Cell number increased on all of the films over 7 days of culture, with the osteoblasts on glass substrates as the positive control also giving the best results, as the commercial glass had been reported to benefit for cell adhesion and spreading, due to its rough surface and hydrophilicity^16, 27^. The proliferation of osteoblasts on P-PUUs substrates was significantly greater (p < 0.05) than that on PDLLA substrates after 3 days of culture, except for the proliferation of osteoblasts between PDLLA and P-PUU-1 at 3 days of culture. Meanwhile, there was a significant difference (p < 0.05) during the proliferation of osteoblasts on the P-PUUs after 3 days of culture, and a continuous improvement in the proliferation of osteoblasts was observed with increasing the PP amount in the P-PUUs.

### Alkaline phosphatase (ALP) activity and extracellular calcium (EC) production

**Figure 6:**
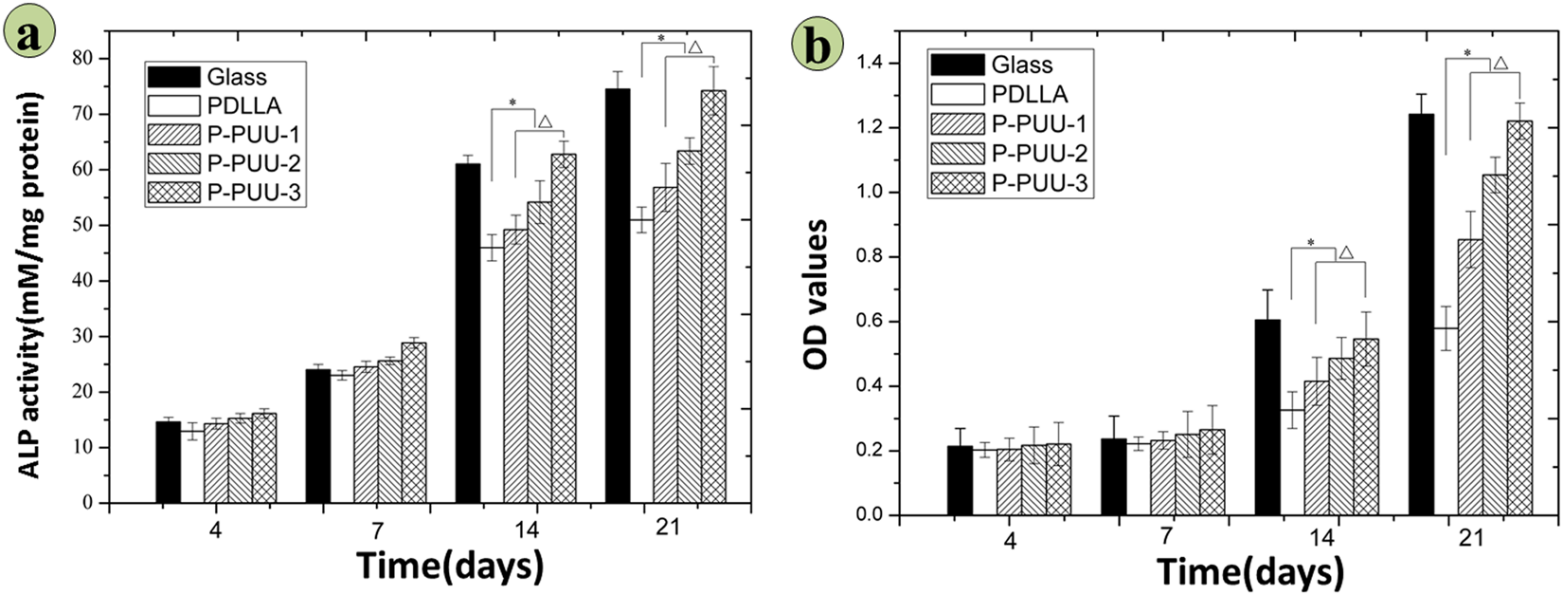
Osteogenic differentiation of osteoblasts on different substrates: (**a**) ALP activity after incubation for 4, 7, 14, and 21 days; (**b**) Extracellular calcium production after incubation for 4, 7, 14, and 21 days (# means P < 0.05, compared with glass groups; * means P < 0.05, compared with PDLLA groups; Δ means P < 0.05, compared with P-PUUs groups).

The ALP activity of osteoblasts seeded on different substrates after 4, 7, 14, and 21 days of culture is shown in Fig. 6a and the ALP-positive areas of osteoblasts seeded on different substrates after 14 days of culture are stained and shown in Fig. S1. The ALP activity of ROBs on P-PUU substrates was significantly greater (p < 0.05) than on PDLLA substrates at 14 and 21 days of culture, whereas there was no statistical difference in ALP activities with cells cultured on P-PUUs and PDLLA substrates at 7 days of culture. Moreover, after 14 and 21 days of culture, the ALP activity sharply increased with increasing PP levels (p < 0.05). In particular, at 21 days culture, the ALP activity of osteoblasts on P-PUU-3, with the highest amount of PP, almost reached the level of ALP activity of osteoblasts on glass.

As is shown in Fig. 6b, calcium deposition were quantitatively detected with alizarin red after 4, 7, 14, and 21 days of culture. In this case, the calcium deposition was certainly generated by biological mineral deposition but not physicochemical precipitation, due to the surroundings of cell culture medium (not the supersaturated SBF solution) and acidic degradable polymeric substrates, which were insufficient conditions for physicochemical precipitation of calcium salts^12–14^. Accordingly, the results of alizarin red staining could be used to express EC secretions by osteoblasts, further revealing the osteogenic differentiation of osteoblasts cultured on polymeric substrates together with ALP activity.

The trends in the variation of EC secretions of all samples were similar to those for the ALP activity. Within the first 7 days of incubation, osteoblasts on various substrates produced EC at a low rate with no significant differences (P < 0.05). However, at 14 and 21 days of culture, the EC secretion of osteoblasts cultured on P-PUUs was significantly greater (p < 0.05) than that on PDLLA substrates, and the EC secretion increased sharply with increasing PP levels (p < 0.05). The highest value of EC secretion of P-PUUs was observed with P-PUU-3 groups on day 21, similar to the value of EC secretion on glass samples. These results were further confirmed by the stained images of EC mineralization by osteoblasts cultured on substrates for 21 days shown in Fig. S2.

### Osteogenesis-related gene expressions

**Figure 7:**
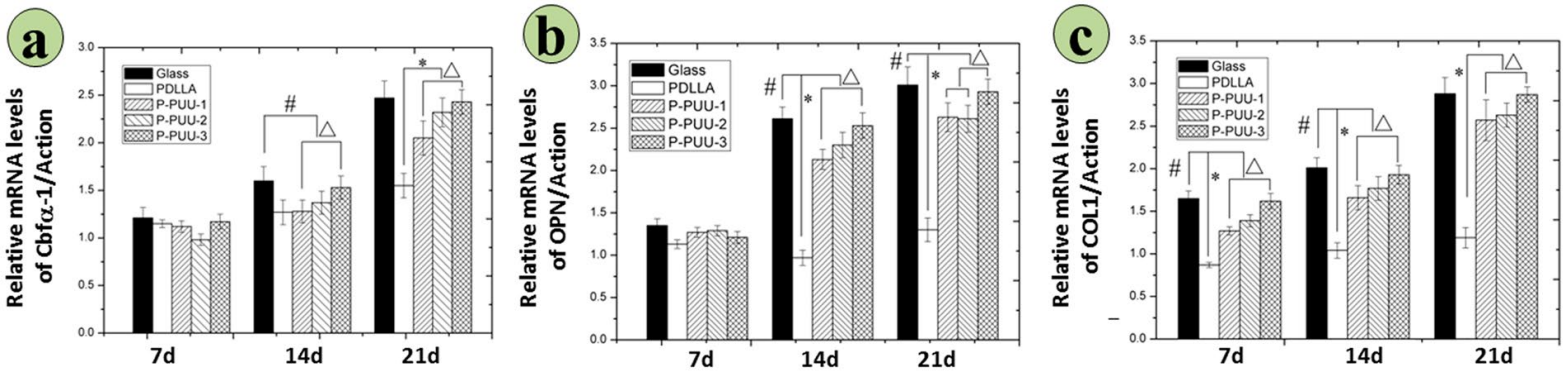
Osteogenesis-related gene expressions on different substrates by RT-PCR analysis after incubation for 7, 14, and 21 days: (**a**) Cbf-α-1; (**b**) OPN; (**c**) COL 1. (# means P < 0.05, compared with glass groups; * means P < 0.05, compared with PDLLA groups; Δ means P < 0.05, compared with P-PUUs groups).

Figure 7 summarizes the osteogenesis-related gene expressions of Cbfα-1, OPN and COL1 at 7, 10, and 14 days of incubation with osteoblasts on various substrates. In general, the expression folds of the three target genes on various substrates were low and irregular after 7 days of culture, except for COL1 of osteoblasts, with a significant difference (p < 0.05) among various samples. However, at 14 and 21 days of culture, the expression and activation of Cbf α-1, OPN and COL1 were up-regulated. The expression folds of the three osteoblast genes on P-PUUs were significantly greater (p < 0.05) than those on PDLLA, except for the expression of Cbf α-1 between PDLLA and P-PUU-1 at 14 days of culture. Moreover, the expression folds sharply increased with increasing PP levels (p < 0.05), except for the expression of OPN between P-PUU-1 and P-PUU-2 at 21 days of culture.

## Discussion

It has been recognized that a relatively high local pH is necessary during the bone formation process^28^, as the optimum activity of alkaline phosphatase (ALP) occurs at pH 8.5 instead of the nominal ‘physiological’ value of 7.4^29^. Acidic degradable biomaterials (degraded with release of acidic products) further acidify the microenvironment, and will lower the local pH. Therefore, it is extremely valuable to further explore the variation of microenvironment pH of acidic degradable biomaterials and whether acidic degradable biomaterials are suitable for bone regeneration.

In the present study, P-PUU was as an example of an acidic degradable biomaterial. They were obtained with a gradient of PP by regulating the molar ratio of diol: HDI: PP from 1:1.1:0.1 to 1:1.4:0.4 as described previously^6, 25^. As indicated in Fig. 1a, introducing two PP segments into the P-PPU backbone improves the controllable degradation and mechanical behavior, in agreement with previous studies^6, 25^. Moreover, uniformly polymeric substrates were successfully prepared using a spin-coating technique with gradient N% and tunable hydrophilicity, ensuring their surface morphology was consistent.

It is known that there are two main factors that impact the interfacial pH of biodegradable materials with cultured medium. One is the surroundings medium, which has a buffering ability to maintain suitable conditions for cell growth. The other is the release of acidic or alkaline products during the degradation process of materials, which are not immediately buffered by the medium^30, 31^. For P-PUUs and PDLLA, it can be observed in Fig. 4a that before 7 days of culture, the interfacial pH of every point is similar to the medium pH, but after 7 days of culture, the interfacial pH of every point was lower than the medium pH. This suggests that the polymeric substrates begin to degrade after culturing for 7 days with medium, when the acidic products from P-PUU and PDLLA will decrease the interfacial pH of the polymeric substrates. Moreover, introducing alkaline segments to the backbone of polymeric substrates can effectively relieve the shortcoming of the acidic conditions during the degradation process of polymeric biomaterials, which is consistent with the results of the bulk degradation behavior of polymers, as shown in our previous report^6, 21^.

Interestingly, when polymeric substrates co-culture with osteoblasts, the variation of the interfacial pH of polymeric substrates shows an upward tendency (Fig. 4b), different to the tendency of polymeric substrates without osteoblasts (Fig. 4a). Furthermore, with an increase in the amount of PP, the interfacial pH will be dramatically improved, and at 21 days of co-culture, the interfacial pH of the glass substrate is the greatest, almost approaching the optimum value for osteoblast growth^13^. As a result, another important factor for markedly improving the interfacial pH must be the osteoblasts themselves, which can contribute to their optimum survival microenvironment by secreting alkaline products^32, 33^, such as mineral calcium salts.

**Figure 8:**
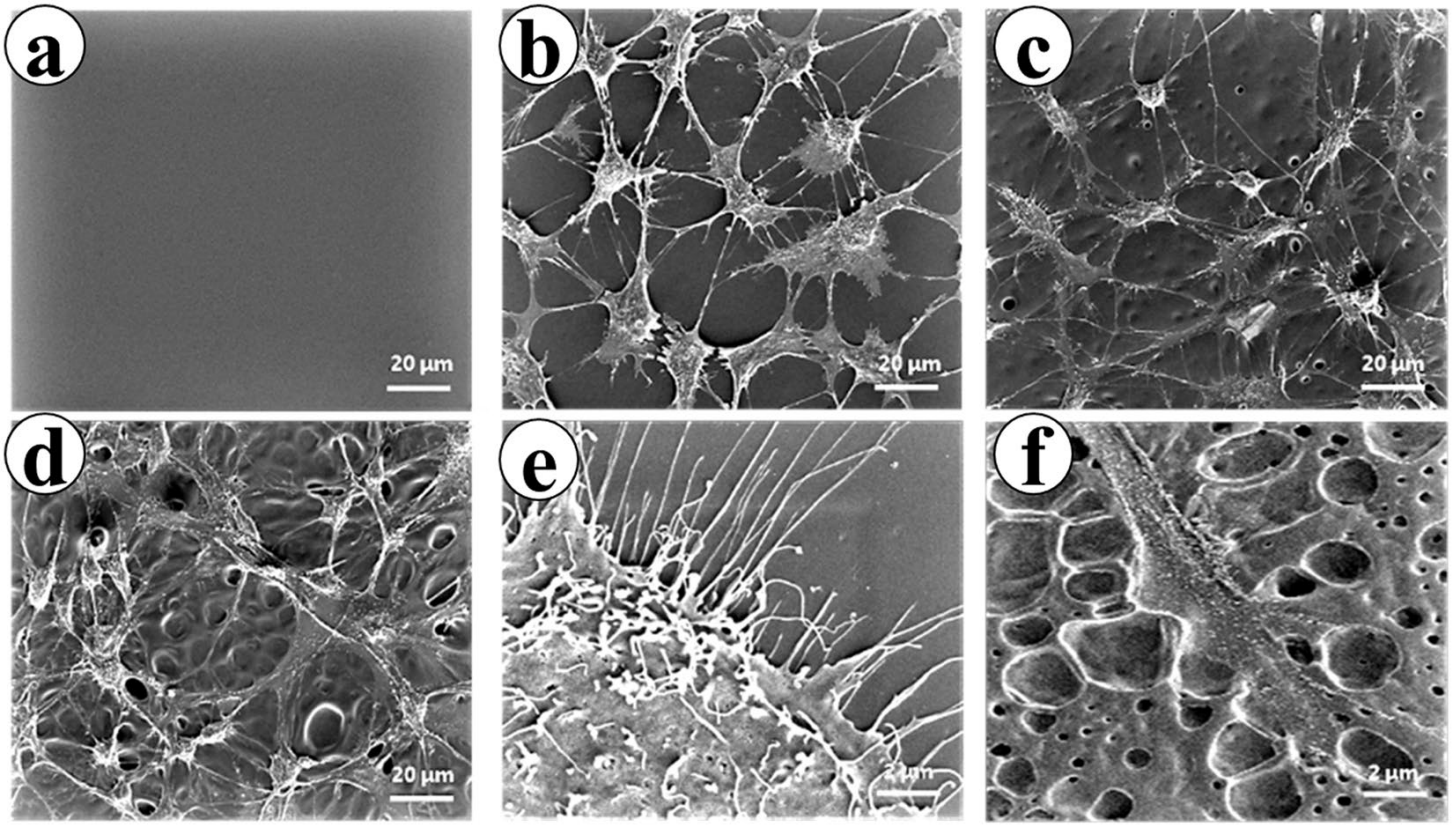
The variation of surface morphologies of P-PUU-2 substrates by incubation with osteoblasts for 7, 14, or 21 days. (**a**) The P-PUU-2 films without culturing; (**b**) The P-PUU-2 films with osteoblasts cultured for 7 days; (**c**) The P-PUU-2 films with osteoblasts cultured for 14 days; (**d**) The P-PUU-2 films with osteoblasts cultured for 21 days; (**e**) Cellular secretion after culturing for 21 days; (**f**) Plentiful micropore formation on films by degrading with cells after culturing 21 days. (Magnification: (**a**–**d**) 1000×; (**e**,**f**) 10000×).

In order to further investigate the interaction of the polymeric substrates and the osteoblasts in their co-culture system, the behavior of the P-PUU-2 substrate with osteoblasts was detected by SEM as shown in Fig. 8a–f. The P-PUU-2 substrate before culturing was confirmed to have smooth surface (Fig. 8a), while the P-PUU-2 substrate cultured with osteoblasts for 7, 14, and 21 days are shown in Fig. 8b–d, respectively. From Fig. 8b,d, it can be seen that more and more micropores appear in the interfaces of polymeric substrates, owing to the degradation behavior of P-PUUs in the medium, and simultaneously the well growth of osteoblasts with proliferation and differentiation were occurring. In particular, it can be seen in Fig. 8e that the cellular secretions were so abundant that they covered the surface of polymeric substrate, and in Fig. 8f that the polymeric substrates were intensely degraded with rough surfaces after 21 days of co-culture. Therefore, both the degradation products from the materials and the secretions from cells play a vital role in regulating the interfacial pH in the co-culture. Without cells, the interfacial pH is mainly affected by the release of the material substrates. On the other hand, when cells are seeded on substrates, the cell can construct the microenvironment by secreting cellular metabolites to satisfy its own survival^32, 33^, combined with the behavior of the biomaterial degradation products affecting the microenvironment pH. For acidic degradable polymeric biomaterials, the release of acidic degraded products obviously causes a rapid decrease in the interfacial pH, but this can be relieved by introducing alkaline segments, and eliminated by the secretions of osteoblasts for proliferation, differentiation and mineralization.

**Figure 9:**
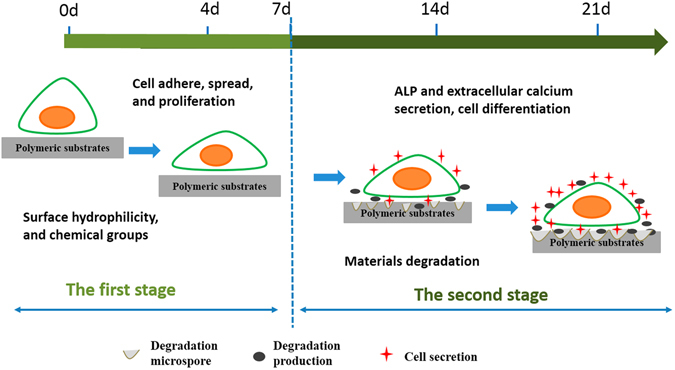
Schematic to show the balance between polymer degradation and cell growth: (**a**) in the first stage, surface hydrophilicity and chemical groups of polymeric substrates play key roles to promote cell adhesion, spread and proliferation; (**b**) in the second stage, the polymeric substrates begin to degrade, affecting the interfacial pH and the secretions from cells, which contribute to the enhanced effect on osteogenic differentiation of osteoblasts.

When an acidic degradable polymeric biomaterial is implanted for bone regeneration in the body, the host cell biological responses to the bone graft may be mainly dependent on the cell/material interaction, which can be regulated by material characteristics such as surface topography^34^, surface hydrophilicity^35, 36^, and chemical composition^37^. Luo *et al*. reported that substrates with moderate amino groups could improve protein adsorption, integrin binding and focal adhesion assembly, which are correlated with good adhesion and spreading of osteoblasts^37^. Groth *et al*.^35^ and Iwata *et al*.^36^ independently demonstrated that cell adhesion and spreading were enhanced on moderately wettable surface, while hydrophobic or non-ionic hydrophilic surfaces inhibited interaction with cells. In this study, the hydrophilicity (Fig. 2) and N content (Fig. 3) of P-PUUs were shown to improve with an increasing number of PP segments, but the surface morphology (Fig. 2) of P-PUUs and PDLLA was similar. In addition, in the early stages of co-culture, the interfacial pH of all samples changed only slightly, and was mostly around pH 7.4 (Fig. 4). This is because the polymers had not begun to degrade at the first 7 days. Therefore, as is shown in Fig. 9, in the early stages of cellular responses of cell morphology and proliferation, osteoblasts prefer to adhere, spread and grow on the P-PUU substrates with more PP units. This is because the hydrophilic surface and the moderate N% in P-PUUs are the contributing factors in the promotion of early stages of cellular responses.

After 7 days of co-culture, the substrates of the polymers begin to degrade, and the osteoblasts also move to the second stage of osteogenic differentiation (Fig. 9). ALP activity and the EC secretion were early and late markers of osteogenic differentiation^38^, respectively. Compared to PDLLA, P-PUUs can promote osteoblast differentiation, and with an increase in PP levels, P-PUUs give a better opportunity for stimulating osteoblast differentiation, which is also confirmed by the expressions of osteogenesis-related genes, Cbfα-1, OPN and COL1. The interfacial pH showed a rapid decrease (Fig. 4a) with the release of degraded products, but this only plays a minor role in the effect of osteoblast growth and differentiation. In general, the release of alkaline PP fragments is capable of neutralizing excessive acidic products from the degradation of PDLLA segments, and consequently the interfacial pH would improve with an increase of PP levels, which is beneficial for creating a suitable microenvironment facilitating the differentiation of osteoblasts. Simultaneously, the osteoblasts, co-cultured with the more suitable interfacial pH of materials, would secrete more proteins and minerals, consisting of ALP and EC (Fig. 6), which would positively regulate the interfacial pH of the materials (Fig. 4b). As a result, although P-PUU is a polyester-based polymer, and the degraded products present acidity, it can be designed as an ideal biomaterial for bone regeneration.

## Conclusion

P-PUUs substrates with gradient PP amounts, and PDLLA substrates were used to investigate whether acidic biodegradable materials with an acidic interfacial pH are suitable to use as scaffold materials for bone regeneration. The surface morphology of these substrates was similar, but the surface hydrophilicity and the surface N% could be improved with an increase in the PP levels. Investigation of the interfacial pH of the substrates with or without osteoblasts demonstrated that the release of degraded products from the polymers caused a rapid decrease in the interfacial pH, but this could be relieved by the introduction of alkaline segments, and eliminated by the secretions of osteoblasts for proliferation, differentiation and mineralization. Furthermore, the osteoblast behavior, including morphology, cytoskeleton, proliferation, ALP activity, EC production, and osteogenesis-related gene expressions, on polymeric substrates revealed that P-PUUs could promote cell growth and osteogenic differentiation of cells better than PDLLA. The promotion ability of P-PUU-3 (with the most PP units) almost reached the level of the positive control glass groups, resulting from the hydrophilic surface and the moderate N% in P-PUUs, which are contributing factors in the promotion of the early stages of cellular responses. The interfacial pH between the cells and the substrates was also a contributing factor to the enhanced effect on osteogenic differentiation of osteoblasts. Accordingly, it can be concluded that acidic biodegradable polymeric biomaterials are effective scaffold materials for bone generation when they are designed with appropriate alkaline segments.

## Experimental section

### Preparation of polymeric substrates

P-PUUs were obtained as described previously^25^. The synthesis route and chemical structures are illustrated in Fig. 1a. The soft segments are tri-block macrodiols of poly (D, L-lactide) and piperazine (PDLLA-PP-PDLLA diol) with a number-average molecular weight (*M*_*n*_) of 3592 (determined by ^1^H NMR spectra). PDLLA-PP-PDLLA diol was first reacted with hexamethylene diisocyanate (HDI) at 75 °C for 3 h with Sn(Oct)_2_ (0.75% with respect to the macrodiol, mol/mol) as the catalyst, and then the piperazine (PP) chain-extender was added for 2 h at 30 °C. The P-PUUs were divided into three groups according to the molar ratios of diol/HDI/PP in the reaction feed, i.e. P-PUU-1, P-PUU-2, P-PUU-3, with diol/HDI/chain extender = 1/1.1/0.1, 1/1.2/0.2 and 1/1.4/0.4, respectively. The molecular weights of P-PUUs were determined by gel permeation chromatography with multi-angle laser light scattering (laser photometer Dawn EOSTM, Wyatt Technology Corporation, California, USA).

PDLLA (*M*_n_ = 58,430, PDI = 1.14) was synthesized as the control via ring-opening polymerization of D, L-lactide as published previously^39, 40^. PDLLA was chosen due to the similar chemical structure between PDLLA and the soft segments of P-PUUs, and further its degradation products are acidic. The properties of polymers used in this set of investigations are summarized in Table 1.

The polymer substrates for the subsequent experiments in this study were prepared as shown in Fig. 1b ^41^. Firstly, the polymers (PDLLA or P-PUUs) were dissolved in chloroform (CHCl_3_) to give solutions of concentration 40 mg/mL, and then the polymer solutions were filtered through a 0.22 µm filter to sterilize and remove the impurities. Next, 50 µL of polymer solution was uniformly dropped onto the clean glass (diameter = 14 mm) using a spin-coating technique (Chemat Scientific, Shanghai, China) under aseptic conditions. The transparent polymer substrates were obtained by slowly evaporating the solvent from the covered glasses at 25 °C for 96 h. The clean glass without polymeric coat was also used as a positive control, due to its non- degradability. All substrates were further sterilized by ultraviolet irradiation for 30 min before co-culturing with cells.

### Surface morphology by AFM

Atomic force microscope (AFM) was used to observe the surface morphologies of the different substrates (glass, PDLLA and P-PUUs). All images were captured using otespa probe at 1 Hz frequency in a non - contact mode at room temperature. The area of image projected surface was 4 μm^2^.

### Static water contact angles of polymeric substrates

Static water contact angles of substrates were assessed using a Model 200 video-based optical system (Future Scientific Co. Taiwan, China) at ambient humidity and temperature when distilled water was used as the medium (5 μL per drop). Each data point represents the average and standard deviation of ten measurements on each specimen for statistical accountability.

### Nitrogen content of polymeric substrates measured by XPS

The XPS spectra of substrates were collected using a XSAM 800 photoelectron spectroscope (Kratos, Manchester, UK) with an Al Kα X-ray source (*hν* = 1486.6 eV) under ultra-high vacuum conditions (2 × 10^−7^ Pa). The binding energy scale was set with N-C bonds at 399.8 eV. Data analysis was carried out with a commercial software package (XPS PEAK, Version 4.1) and the nitrogen content ratio of polymer substrates was calculated using equation (1).

1 $$N \% =N/(C+O+N)\times 100 \% $$

where N, C and O represent the nitrogen content, the carbon content and the oxygen content of polymer substrates, respectively.

### Interfacial pH of polymeric substrates

The interfacial pH of polymeric substrates with or without osteoblasts were measured using a flat membrane microelectrode (MI-406, Microelectrodes, Bedford, NH) with a separate reference electrode (MI-401, Microelectrodes) as published previously^13, 14^, while a glass slide with non-degradability was used as a positive control.

The interfacial pH of the polymeric substrates and glass cultured in medium without cells was measured to evaluate the variation of the interfacial pH of the polymeric substrates. The polymeric substrates were immersed in 1 mL of DMEM/F12 supplemented with 10% heat-inactivated fetal calf serum, penicillin (100 U/mL), streptomycin (100 µg/mL and 0.5% L-glutamine, and maintained in a controlled atmosphere (5% CO_2_/95% air, 37 °C). The medium was renewed every 2 days, and the pH at the solid-liquid interface was measured at 1, 4, 7, 10, 13, 16, and 19 days at five random locations using the microelectrode. In addition, as the electrode was withdrawn, pH values at positions 3 mm above that surface were recorded.

Furthermore, to explore the variation of interfacial pH of polymeric substrates with cells, primary rat osteoblasts (ROBs) were seeded on polymeric substrates at a density of 1 × 10^4^ cells/well, then the methods of culture and test for the above experiments of the polymeric substrates without cells were followed. Here, the measure points were reduced to four times at 3, 7, 14 and 21 days to reduce the risk of cell contamination.

### Osteoblast culture

ROBs were isolated from minced Sprague-Dawley rat calvarial chips (three different SD rats, born within 3 days) as described previously^42^, and this operation was guided under the Ethics Committee for Animal Research, Shenzhen Institutes of Advanced Technology, Chinese Academy of Sciences and the ethical approval number for this investigation was SIAT-IRB-150302-YYS-RCS-A0102. Cultures were initiated in DMEM/F12 supplemented with 10% heat-inactivated fetal calf serum, penicillin (100 U/mL), streptomycin (100 µg/mL and 0.5% L-glutamine and maintained in a controlled atmosphere (5% CO_2_/95% air, 37 °C). The medium was renewed every 2 days. The third to fifth passage cells were used for all experiments. All samples were placed in 24-well tissue culture plates for cell seeding and culturing. Cells were seeded onto polymer substrates at a density of 1 × 10^4^ cells/well for all experiments, except for specified descriptions.

### Osteoblast morphology

Osteoblast morphology on different substrates was observed by means of fluorescent staining as established previously^26^. For staining^43^, briefly, cells were cultured for 24 h with 3.7% formaldehyde for 20 min at 4 °C, followed by rinsing with PBS (pH 7.4) solution and permeabilizing with 0.2% Triton X-100 for 2 min at 4 °C. Then, cells were stained with 5 U/mL rhodamine-phalloidin (Invitrogen Co., California, USA) at 4 °C overnight to stain their cytoskeleton and with 5 g/mL Hoechst 33258 (Sigma-Aldrich, California, USA) for 1 min to stain their nuclei. The fluorescent images were observed with a LSM 510 META laser confocal microscope (Carl Zeiss, Heidenheim, Germany). Cell spreading area was measured by ImageJ (NIH) using the active contours algorithm (n = 200).

The Live/dead staining was utilized to perform the primary cell behaviors on different substrates. After seeding ROBs on polymeric films for 24 h of culture, the samples was stained with Live/dead viability/cytotoxicity kit for 30 min at 25 °C and the fluorescent images were observed with a fluorescence microscopy (BX53, Olympus, Tokyo, Japan).

The variation of surface morphologies of P-PUU-2 during co-culturing with osteoblasts were observed by Scanning electron microscopes (SEM). The co-cultured samples were prepared using the following procedure: P-PUU-2 was co-cultured for 7, 14 and 21 days. At the time points of 7, 14 and 21 days, each polymeric substrate plus osteoblasts was fixed with 4% (wt/v) paraformaldehyde at 4 °C for 30 min, and then washed thrice in PBS. Subsequently, all samples were dehydrated in graded ethanol (V_ethanol_/V_distilled water_ = 30%, 50%, 70%, 80%, 90%, 95% and 100%) for 10 min. Finally, the samples were freeze-dried before use. All the samples were sputter-coated with gold and observed with a Nova NanoSEM 450 (FEI, Oregon, USA).

### Osteoblast proliferation

The relative cell viability and proliferation was determined using a methylthiazolyl tetrazolium assay (MTT assay)^44^. Briefly, ROBs were cultured on different substrates for 1, 3, 5, and 7 days. At every culture time point, quantities of 500 µL DMEM and 100 µL MTT solution (5 mg/mL in PBS) were added to each sample and incubated for 4 h at 37 °C. The blue formazan product was dissolved by adding 0.5 mL DMSO and transferred to a 96-well plate. The absorbance at 570 nm was measured using a Bio-Rad 550 spectrophotometric microplate reader, and the obtained results were linear with the living cell number.

### Alkaline phosphatase (ALP) and extracellular calcium (EC)

ROBs were seeded on polymeric substrates at a density of 1 × 10^5^ cells/well to determine the levels of ALP activity and EC.

For the ALP assay^45^, after 7, 14, and 21 days of culture, the cells were washed thrice with PBS and lysed in 0.2 vol. % Triton X-100. The ALP activity was determined using a colorimetric assay with an ALP reagent containing p-nitrophenyl phosphate (p-NPP) as the substrate (Beyotime, Shanghai, China). The absorbance of p-nitrophenol was monitored at 405 nm. The intracellular total protein content was determined using the MicroBCA protein assay kit (Thermo Pierce, California, USA), and the ALP activity was normalized to the total protein content. For ALP staining, the samples cultured for 14 days were rinsed twice with PBS, fixed by 4% (wt/v) paraformaldehyde at 4 °C for 30 min, and rinsed with ultra-pure water for 45 s. Then the samples were stained with BCIP/NBT alkaline phosphatase color development kit (Beyotime, Shanghai, China) followed the manufacturer’s protocol and pictures were taken by microscopy (BX 53, Olympus, Tokyo, Japan).

The extracellular calcium (EC) mineralization by osteoblasts on the samples was evaluated using Alizarin Red staining method^45^. After 7, 14, and 21 days of culture, the osteoblasts were washed thrice with PBS and fixed in 75% ethanol for 1 h. The samples were stained with 40 mM Alizarin Red in distilled water (pH = 4.2) for 10 min at room temperature. Afterwards, the samples were washed with distilled water until no color appeared, and pictures were taken by microscopy (BX 53, Olympus, Tokyo, Japan). For the quantitative analysis, the stain was dissolved with 10% cetylpyridinium chloride in 10 mM sodium phosphate (pH = 7.0) and the absorbance was monitored at 540 nm.

### Osteogenesis-related gene expressions

ROBs were seeded onto polymeric substrates at a density of 1 × 10^5^ cells/well, and osteoblasts were collected after incubating 7, 14, and 21 days, and the total cellular RNA was extracted according to the instructions of the RNA extract kit (Biotek Co., Vermont, USA). RNA purity was determined from absorptions at 260 and 280 nm. RNA integrity was assessed as the 28s/18s rRNA ratio after electrophoresis on 1.2% agarosegels. The reverse transcription was performed according to the instructions of the RNA reverse kit (Fermentas Co., Ontario, Canada). PCR was performed in 42 cycles at the following reaction conditions: denaturing at 95 °C for 30 s, annealing at 58 °C for 15 s, and extension at 65 °C for 1 min. A final 10 min extension step was performed to ensure the completion of the products. All PCR products were verified on 1.2% agarose gel stained with ethidium bromide. The intensities of specific bands were determined by densitome-try. The genes of collagen I (COL 1), osteopontin (OPN) and core-binding factor α-1 (Cbfα-1) were the target genes related to osteogenesis, while the β-actin gene was used as a control in the RT-PCR assay. Table 2 listed the primer sequences of the tested genes.

**Table 2:** Oligonucleotide primers for gene expression analysis in RT-PCR.

Gene	Primer sequence (F = forward, R = reverse)	Length of product/bp	Accession no.
COL I	F5′GTCTTCCTGGTGAATTCGGT3′	102 bp	NM_007743
	R5′TTCCAATAGGACCAGAAGGG3′		
OPN	F5′CTTTCACTCCAATCGTCCCTAC3′	165 bp	NM_009263
	R5′CCTTAGACTCACCGCTCTTCAT3′		
Cbfα-1	F5′AGTTCCCAAGCATTTCATCC3′	145 bp	AF_010284
	R5′GGCAGGTACGTGTGGTAGTG3′		
β-actin	F5′GGTCATCACTATTGGCAACG3′	72 bp	NM_007393
	R5′GGCAGGTACGTGTGGTAGTG3′		

Collagen I (COL 1), osteopontin (OPN) and core-binding factor α-1 (Cbfα-1).

### Statistical analysis

Multiple samples (n = 3 to 6) were gathered in each experiment and the data were expressed as mean ± standard deviation (SD). Each experiment was repeated independently for three times to assure reproducibility. Statistical differences among the groups were determined by one way ANOVA. Results were considered statistically significant when p values were <0.05.

## Electronic supplementary material

Supplementary Information
