# Supplementary material for: The interfacial pH of acidic degradable polymeric biomaterials and its effects on osteoblast behavior
Source: Sci Rep. 2017 Jul 28;7:6794. doi: 10.1038/s41598-017-06354-1 (PMC5533751; doi:10.1038/s41598-017-06354-1)
Supplement: Supplementary file 1 — Supplementary Information [file 41598_2017_6354_MOESM1_ESM.doc]

Supporting information

**The interfacial pH of acidic degradable polymeric biomaterials and its effects on osteoblast behavior**

Changshun Ruan ^1, +^, Nan Hu ^2, +^, Yufei Ma ^1^, Yuxiao Li ^3^, Juan Liu^1^, Xinzhou Zhang ^2,*^, and Haobo Pan ^1,*^

^1^Center for Human Tissue and Organs Degeneration, Institute of Biomedicine and Biotechnology, Shenzhen Institutes of Advanced Technology, Chinese Academy of Sciences, Shenzhen, Guangdong 518055, China;

^2^Key Renal Laboratory of Shenzhen, Department of Nephrology, Shenzhen People’s Hospital, The Second Clinical Medical College of Jinan University, Shenzhen, Guangdong 518020,China;

^3^Department of Biochemistry and Molecular Biology, Southwest Medical University, Luzhou, Sichuan 646000, China;

**Corresponding Authors**

* E-mail: [xin.zhou@medmail.com.cn](mailto:xin.zhou@medmail.com.cn) (Xinzhou Zhang)

* E-mail: [hb.pan@siat.ac.cn](mailto:hb.pan@siat.ac.cn) (Haobo Pan)

^+^ These authors contributed equally to this work.

**
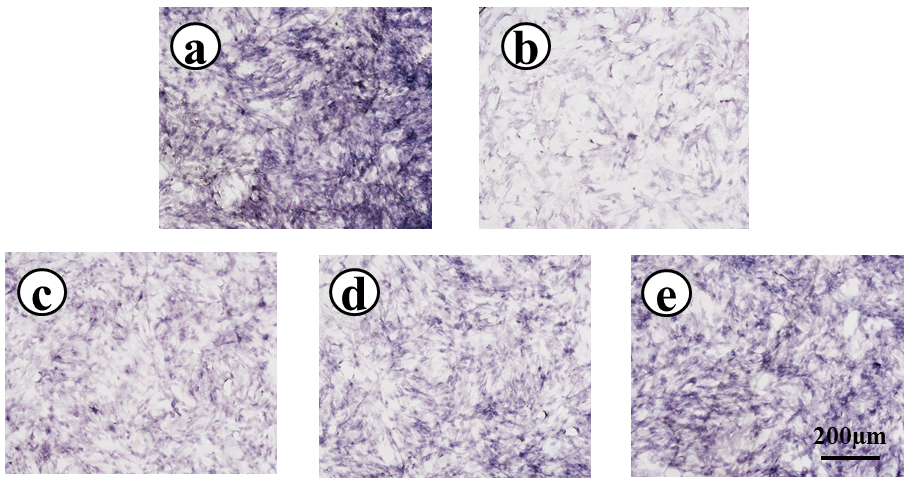
**

**Figure S1. ALP activity assay of osteoblasts cultured on the substrates for 14 days.** a) Glass; b) P-PUU-1;c) P-PUU-2;d) P-PUU-3.

**
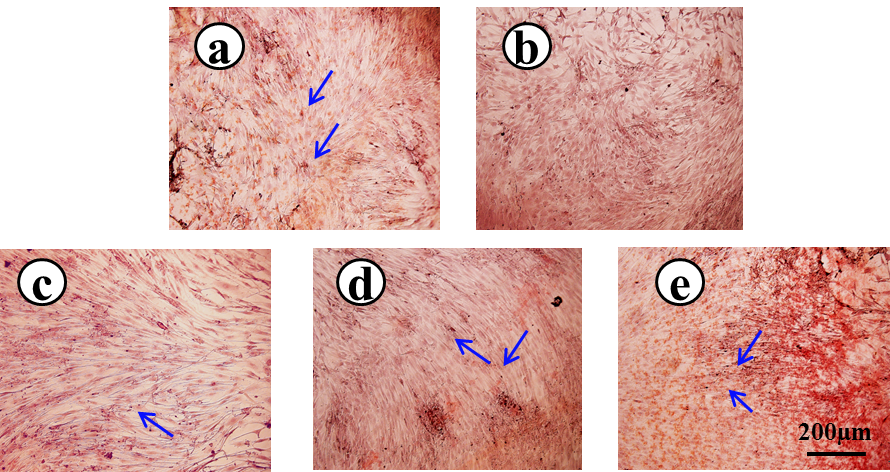
**

**Figure S2.** **Extracellular calcium production of osteoblasts cultured on the substrates for 21 days**. a) Glass; b) P-PUU-1;c) P-PUU-2;d) P-PUU-3. Small calcium nodes are marked by blue arrows
